# Clinicopathological characteristics and treatment outcomes of Chinese patients with genitourinary embryonal rhabdomyosarcoma

**DOI:** 10.1186/s12957-015-0574-x

**Published:** 2015-05-28

**Authors:** Xiao-kai Zhan, Sen Zhang, Bang-wei Cao, Jin-wan Wang, Jun-ling Li, Yong-kun Sun, Wen Zhang, Lin Yang, Ai-ping Zhou, Yi-he bali Chi, Ye-xiong Li, Jian-hui Ma, Chang-ling Li

**Affiliations:** Department of Oncology, Beijing Friendship Hospital, Capital Medical University, Beijing, China; Department of Medical Oncology, Cancer Institute and Hospital, Chinese Academy of Medical Sciences, Beijing, China; State Key Laboratory of Bioactive Substances and Functions of Natural Medicines, Institute of Materia Medica, Chinese Academy of Medical Sciences and Peking Union Medical College, Beijing, China; Department of Radiation Oncology, Cancer Institute and Hospital, Chinese Academy of Medical Sciences, Beijing, China; Department of Urological Surgical Oncology, Cancer Institute and Hospital, Chinese Academy of Medical Sciences, Beijing, China

**Keywords:** Genitourinary embryonal rhabdomyosarcoma, Chinese patients, Treatment, Chemotherapy

## Abstract

**Background:**

Genitourinary embryonal rhabdomyosarcoma is rarely reported in China. This retrospective analysis aimed to characterize the clinicopathologic features and treatment outcomes of genitourinary embryonal rhabdomyosarcoma in a sample of Chinese patients.

**Methods:**

Basic demographic and clinical data of 29 patients, who were diagnosed with genitourinary embryonal rhabdomyosarcoma between January 2000 and December 2011, were retrieved and analyzed.

**Results:**

In these patients, 25 were males and 4 were females with a median age of 12 years. Paratesticule was the most common lesion site, followed by the prostate, bladder, and vagina. The median tumor size was 5.80 cm. Six patients had clinically positive regional nodes. At the initial diagnosis, patients had a metastatic disease. According to the TNM staging classification for the IRS-IV, phase I lesions were detected in ten cases, phase II lesions in six cases, phase III lesions in four cases, and phase IV lesions in nine cases. The median survival of all patients was 63 (range from 6 to 118) months. The 1-, 3-, and 5-year survival rates for these patients were 93%, 83%, and 52%, respectively. Multivariate analyses demonstrated that staging and anemia were significant predictors of prognosis.

**Conclusions:**

Our findings suggest that metastasis predicts a poor prognosis. Chemotherapy played an important role in comprehensive treatment. Palliative and neo-adjuvant chemotherapy could increase median survival time.

## Background

Rhabdomyosarcoma is a rare disease, accounting for 3% of all childhood cancers. A total of 350 new cases occur each year estimated in the USA [1]. Rhabdomyosarcoma can develop in almost any part of the body, and in up to 29% of the cases, it arises in genitourinary organs [2], including the bladder, prostate, vagina, and paratesticule. Alveolar and embryonal rhabdomyosarcomas are the two most common histological subtypes of rhabdomyosarcoma. Approximately, 90% of genitourinary rhabdomyosarcoma cases in non-Chinese patients are embryonal [3]. Little is known about genitourinary embryonal rhabdomyosarcoma in the Chinese population. This retrospective study attempted to characterize the clinicopathological manifestations and evaluate the current treatment outcomes of genitourinary embryonal rhabdomyosarcoma in China.

## Methods

### Patients and ethnic consideration

Medical records of patients, who were diagnosed with embryonal rhabdomyosarcoma and treated at Friendship Hospital in Beijing between January 2000 and December 2011, were reviewed. After exclusion of retroperitoneal and pelvic embryonal rhabdomyosarcomas, 29 cases of genitourinary embryonal rhabdomyosarcoma included in this retrospective analysis. Data on age, sex, primary site, clinical and histopathological features, and treatment outcomes were analyzed.

The study was approved by Beijing Friendship Hospital Ethics Committee and conducted in compliance with the provisions of the Declaration of Helsinki. Informed written consent was obtained from all subjects.

### Diagnosis of genitourinary embryonal rhabdomyosarcoma

The diagnosis was made based on the results of physical examination and various tests (*e.g.*, blood biochemistry test, computed tomography (CT) of the abdomen and pelvis, chest radiography, magnetic resonance imaging (MRI) and bone scanning in all cases, and bone marrow aspiration in some cases). Fine needle biopsy was performed on all patients, and radical surgery was performed in 20 cases. Lymph node involvement was determined by biopsies, surgical specimens, and/or CT scanning.

### Histology and immunohistochemical staining

Immunohistochemical staining was performed to detect desmin- and myogenin-positive cells in all 29 resected tumor tissue specimens. Pathological assessment was performed by two experienced pathologists independently, and findings including histological tumor type, depth of invasion, size, margins, and lymphatic invasion were recorded.

### Treatment and follow-up

The lesion was staged according to the American Joint Committee on Cancer (AJCC) staging system [4]. Of the 29 patients, 27 received chemotherapy. Responses were assessed by computed tomography or magnetic resonance imaging scans based on the standard response criteria referred to as Response Evaluation Criteria in Solid Tumors (RECIST) [5]. Time-to-tumor progression (TTP) is defined as the period between treatment initiation and objective tumor progression, progression-free survival (PFS) is defined as the time from randomization until objective tumor progression or death, and overall survival is defined as the time from diagnosis to the death or last visit were recorded.

Patients were followed up to 30 December 2012 by telephone or mail. At each follow-up, complete information on vital status was censored. No single patient was lost to follow-up.

### Statistical analysis

Survival data were analyzed using the Kaplan-Meier method and the log-rank test. Differences were considered statistically significant when *P* < 0.05. The software SPSS 15.0 (Chicago, IL, USA) was used.

## Results

### Clinicopathological characteristics

A total of 129 patients were diagnosed with embryonal rhabdomyosarcoma, among whom 29 (22.48%) had genitourinary embryonal rhabdomyosarcoma. The basic demographic and clinicopathologic data of these 29 patients are presented in Table 1. Briefly, genitourinary embryonal rhabdomyosarcoma occurred predominantly in males; the primary lesion sites were paratesticule, the prostate, and bladder in male patients but exclusively in the vagina in female patients; genitourinary embryonal rhabdomyosarcoma occurred at a much younger age in female patients than in male patients.

**Table 1:** **Characteristic of patients according to the primary sites**

		**All patients**	**Paratesticular**	**Prostate**	**Bladder**	**Vagina**
		***N*** **= 29**	***N*** **= 10**	***N*** **= 8**	***N*** **= 7**	***N*** **= 4**
Sex						
	Male	25	10	8	7	-
	Female	4	-	-	-	4
Median age, years (range)		12 (2 to 27)	15 (12 to 26)	9 (3 to 27)	6.5 (2 to 19)	3.5 (2 to 20)
Number of patients	<10	14	-	5	6	3
	>10	15	10	3	1	1
Number of patients	<2	16	5	3	5	3
	>2	14	5	5	2	1
Status at diagnosis						
Number of patients	Organ confined	20	7	4	6	3
	Metastatic	9	3	4	1	1
WBC		6.8				
RBC		392	391.5	355	403	444.5
Hb		130	129	126.5	137	137
Anemia		5	1	2	1	1
Plt		197	182	173	223	212
Median tumor size (cm)		5.6 (2.5 to 15)	4.9 (4.0 to 13)	7.0 (3.0 to 15)	6.0 (4.5 to 8.7)	3.0 (2.5 to 5.8)
Number of patients	<5	11	6	2	1	3
	>5	18	4	6	6	1
Regional nodes						
Number of patients	Negative	23	9	7	4	3
	Positive	6	1	1	3	1
IRS staging						
Number of patients	I	10	7	-	-	3
	II	6	-	3	3	-
	III	4	-	1	3	-
	IV	9	3	4	1	1

WBC, white blood cells; RBC, red blood cells; Hb, hemoglobin; IRS, Intergroup Rhabdomyosarcoma Study; Plt, platelet.

### Survival data

**Figure 1:**
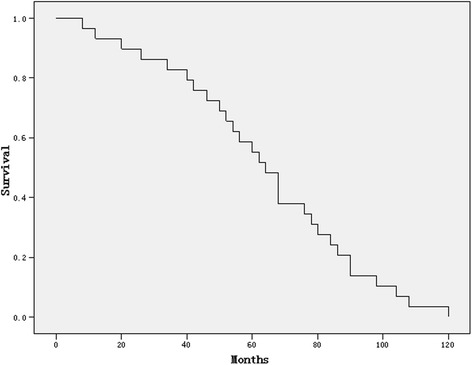
The survival curve of 29 patients diagnosed with embryonal rhabdomyosarcoma.

At the time of the last follow-up, 5 patients were still alive. The overall median, 1-, 3-, and 5-year survival rates of the 29 patients were 63 (range from 6 to 118), 93%, 83%, and 52%, respectively (Figure 1). The survival was not associated with sex, age, and primary sites. Moreover, the survival time of patients with anemia was shorter than those patients without anemia. The detailed data was shown in Table 2.

**Table 2:** **Survival data of the patients**

		**1-year survival rate (%)**	**3-year survival rate (%)**	**5-year survival rate (%)**	**Median OS (months)**	***P*** **value**
Sex	Male	92	88	52	63	
	Female	100	50	50	96	0.479
Age						
	≤10	100	93	53	63	
	>10	86	64	50	66	0.899
Anemia	No	100	96	63	67.33	
	Yes	60	20	0	25	<0.001
Tumor size						
	≤5	100	91	91	88.5	
	>5	89	78	28	52	<0.001
Regional nodes						
	Negative	96	87	61	67	
	Positive	83	67	17	54	0.033
Status						
	Local	100	95	75	76	
	Metastatic	78	56	0	39	<0.001
Primary sites						
	Paratesticular	90	80	70	82	
	Prostate	88	88	38	52	
	Bladder	100	100	43	61	
	Vagina	100	50	50	96	0.349
Sites	Non-B/P	93	71	64	82	
	B/P	93	87	40	59	0.084
Staging						
	I	100	90	90	88	
	II	100	100	83	67	
	III	100	100	25	58	
	IV	78	56	0	39	<0.001

B/P, bladder or prostate; OS, overall survival.

### Prognostic significance of staging and anemia

Given that staging was based on tumor size and regional node involvement, multiple regression analysis was not appropriate to assess tumor size and lymph node involvement as an independent variable; thus, a multivariate analysis was performed. This analysis showed that staging and anemia were independent prognostic factors for genitourinary embryonal rhabdomyosarcoma (Table 3).

**Table 3:** **The staging and anemia confirmed result**

	**B**	**SE**	**Wald**	**df**	**Significance**	**Exp (B)**	**95.0% CI for Exp (B)**	
	**Lower**	**Upper**	**Lower**	**Upper**	**Lower**	**Upper**	**Lower**	**Upper**
Status at diagnosis	.983	1.075	.837	1	.360	2.672	.983	1.075
Staging AJCC	.993	.358	7.704	1	.006	2.699	.993	.358
Anemia	−2.596	1.119	5.380	1	.020	.075	−2.596	1.119

AJCC, American Joint Committee on Cancer; CI, confidence interval.

### Treatment

In the 29 patients, 24 received at least one form of treatment, 3 only received a single treatment and 2 declined treatment. Surgery was performed in 22 cases, radiotherapy in 14 cases, and chemotherapy in 27 cases (Table 4).

**Table 4:** **Performed treatment in 29 patients**

**Number of patients**	**All**	**Paratesticular**	**Prostast**	**Bladder**	**Vagina**
	**29**	**10**	**8**	**7**	**4**
Surgery	20	7	4	6	3
Radiotherapy	6	0	1	4	1
Chemotherapy	27	9	7	7	4
Single modality	7	2	3	1	1
Combined modality	20	7	4	6	3
No treatment	2	1	1	0	0

### Tumor sites

#### Paratesticule

Embryonal rhabdomyosarcoma was diagnosed in the paratesticule where painless scrotal mass was presented in 10 patients. They were all younger than 10 years (median age of 15 years and range 12 to 26 years). Before surgery, CT scanning was performed on all patients, and the histological type was determined by immunohistological staining. At diagnosis, 7 of these patients had a local disease, and the other 3 had a metastatic disease (anemia in one case). The median diameter of tumor was 4.9 cm (range 4.0 to 13 cm), and for an unknown reason, the tumor was biggest in diameter in one anemia case.

Treatment: 5 (50%) patients received radical surgery, including retroperitoneal lymph node dissection (RPLND) and adjuvant chemotherapy (VAC regimen (vincristine, actinomycin D, and cyclophosphamide) or IVA regimen (ifosfamide, vincristine, and actinomycin D)) for 10 to 12 months. The median disease-free survival (DFS) was 29 months. Four (80%) patients developed metastatic disease after adjuvant chemotherapy and were treated with multidrug chemotherapy. Two (20%) patients received neo-adjuvant chemotherapy (VAC for 8 weeks) followed by salvage surgery, and both had partial response and achieved complete tumor resection (R0) with a consolidation chemotherapy for 3 months. Two patients developed recurrent disease and metastatic disease during follow-up period and received salvage chemotherapy. The 2 patients with metastatic disease at the time of diagnosis and 6 patients who developed metastatic disease received palliative chemotherapy. The agents included vincrine, dactinomycin, ifosfamide, etoposide, cyclophosphamide, epirubicin, adriamycin, cisplatin, carboplatin, irinotecan, gemcitabine, and bevacizumab. VAC and IVA were administrated as the first-line chemotherapy. Patients who underwent chemotherapy as the first-line treatment had a response rate (RR) of 20%, and those with relapse had an RR of 80%. The median TTP was 9.5 months, and median survival time was 29 months. One patient who was treated with irinotecan and bevacizumab achieved partial remission. The DFS was 8 months. One patient refused chemotherapy and died of pneumocystis pneumonia 10 months after diagnosis.

Survival: Patients were followed up for a median of 96 months. Three of them were alive at the end of follow-up. The 1-, 3-, and 5-year survival rates were 90%, 80%, and 70%, respectively. Median overall survival was 82 months.

#### Vagina

The median age of the 4 female patients was 3.5 years, and only 1 patient was older than 10 years who presented with irregular menstruation. Before surgery, all had CT scanning. The histological type of the lesion was confirmed by experts. At diagnosis, 3 patients had a local disease and 1 patient had a metastatic disease (anemia in one case). The median diameter of tumors was 3.0 cm (range 2.5 to 5.8 cm). Only one patient had clinically positive regional nodes.

#### Treatment

Two patients received radical surgery, followed by adjuvant chemotherapy (VAC for 12 months). The DFS was 35 and 39 months, respectively, in these two patients. All patients developed a metastatic disease after adjuvant chemotherapy. One of them underwent neo-adjuvant chemotherapy with VAC for 6 weeks. After a partial response, this patient moved onto salvage surgery and radiotherapy with 45 Gy and achieved a DFS of 39 months. One patient was initially diagnosed with a metastatic disease, and two patients developed a metastatic disease. These three patients underwent chemotherapy with multiple drugs including vincrine, ifosfamide, etoposide, teniposide, cyclophosphamide, epirubicin, adriamycin, cisplatin, carboplatin, irinotecan, and gemcitabine. Patients undergoing this first-line multidrug chemotherapy had an RR of 33%, and those with relapse had an RR of 67%. The median survival time of these patients was 38 months.

#### Survival

These patients were followed up for a median of 96 months. At the end of follow-up, only one patient was alive. The 1-, 3-, and 5-year survival rates were 100%, 50% and 50%, respectively. The median overall survival was 96 months.

#### Prostate

Rhabdomyosarcoma was detected in the prostate in 8 patients with a median age of 9 (range 3 to 27) years. The embryonal type of the lesion was confirmed by pathologists. Of these patients, 3 were over 10 years old and presented with gross hematuria, 4 had a local disease, 4 had a metastatic disease, and 2 had anemia. The median diameter of tumor was 7.0 cm (range 3.0 to 15 cm), and only 1 patient had clinically positive regional nodes. Based on the TNM staging classification for the IRS-IV, phase II lesion was in three cases, phase III lesion in one case, and phase IV lesion in four cases.

#### Treatment

Three (37.5%) patients received radical surgery and adjuvant chemotherapy (VAC or IVA for 8 to 10 months) and achieved a median DFS of 17 months. One patient had regional node involvement and underwent neo-adjuvant chemotherapy and salvage surgery. Three patients presented with a metastatic disease at diagnosis, and 3 patients advanced to a metastasis disease. These 6 patients all received multidrug first-line chemotherapy with vincrine, vinorelbine, ifosfamide, etoposide, cyclophosphamide, epirubicin, adriamycin, cisplatin, carboplatin, irinotecan, and gemcitabine and had an RR of 33%, and those with relapses had an RR of 67%. The median survival was 26.5 months. In the very 1 patient with anemia, bone marrow involvement was revealed by bone marrow smears. One patient had a large tumor (10.5 cm in diameter) with metastasis in multiple regional lymph nodes. Despite multiple cycles of chemotherapy, no lesion shrinking was observed and the patient only lived disease-free for 2.5 months that was in a sharp contrast a median overall survival of 22 months in the 8 patients in this category. One patient who did not receive any treatment died 8 months after diagnosis.

#### Survival

The patients were followed up for a median of 48 months, and no one was alive by 30 December 2011. The 1-, 3-, and 5-year survival rates were 88%, 88%, and 38%, respectively. Median overall survival was 52 months.

#### Bladders

Even patients had lesions in the bladder which were confirmed by experienced pathologists as an embryonal type. They were all younger than 10 years (median age of 6.5 years) except that 1 patient was 19 years old who presented with gross hematuria and frequent urination. Before surgery, all these patients underwent CT scanning. Initially, 6 patients were diagnosed with a local disease and 1 with a metastatic disease complicated by anemia. The median diameter of tumor was 6.0 (range 4.5 to 8.7) cm, and 3 patients had clinically positive regional nodes. Based on the TNM staging classification for the IRS-IV, phases II and III lesions were each in three cases and phase IV lesion was in the other case.

#### Treatment

Three of the seven patients received radical surgery, followed by adjuvant chemotherapy (either VAC or IVA for 10 to 12 months) and radiotherapy. The median DFS was 18 months. Two of these three patients developed metastatic disease. Three patients with regional node involvement underwent neo-adjuvant chemotherapy (50 to 60 Gy every 5 to 6 weeks), followed by salvage surgery and radiotherapy. The DFS was 22 months. The very 1 patient with metastatic disease at initial diagnosis together with the 3 patients who developed metastasis disease accepted palliative chemotherapy with a regimen including bleomycin, vincrine, ifosfamide, cyclophosphamide, epirubicin, adriamycin, cisplatin, carboplatin, irinotecan, and gemcitabine. Patients who received the IVA regimen-based chemotherapy had an RR of 25% while those with relapses had an RR of 75%. The median survival was 27 months.

#### Survival

The 7 patients were followed up for a median of 64 months. By 30 December 2011, only 1 patient was alive. The 1-, 3-, and 5-year survival rates were 100%, 100%, and 43%, respectively. Median overall survival was 61 months.

## Discussion

Genitourinary embryonal rhabdomyosarcoma is common in children and young adults. In general, the incidence of this disease is significantly higher in males than in females [6]. The common sites of the lesions include the bladder, prostate, paratesticule, and vagina. Regional lymph node involvement is frequently found in paratesticular rhabdomyosarcoma. Most often, tumor growth results in genitourinary obstruction. Most frequently, genitourinary embryonal rhabdomyosarcoma invades the lung, serosal surface, distant nodes, and bone [4]. In this study, the median age of the subjects was 12 years. The most common lesion site was the paratesticule, followed by the prostate, bladder, and vagina; the median tumor size was 5.60 cm in diameter, and the tumor in the prostate was largest with a median diameter of 7.0 cm; lymph node involvement most frequently occurred in bladder lymph nodes; metastasis occurred most frequently in soft tissue, followed by the lung and bone; subjects in this cohort had a significantly lower survival rate and a significantly higher proportion of metastatic disease as compared with subjects in previous studies [5]. It has to be pointed out, however, 2 patients did not receive any active treatment, and some patients only received chemotherapy or radiotherapy. These might have a negative effect on the survival rate.

Radical surgery used to be the solely primary treatment for genitourinary embryonal rhabdomyosarcoma [3]. However, this approach is associated with a low survival rate (about 20%) [7]. Moreover, the procedure involves a wide extent of resection, often resulting in normal tissue damage and organ (especially urogenital tract) dysfunction [8]. Radiation therapy is another treatment option for genitourinary embryonal rhabdomyosarcoma; it may achieve a local control of tumor growth in patients who have undergone an initial surgical resection but fails to improve patient survival substantially. Compiling clinical data have come to realize that rhabdomyosarcoma is a microscopic metastatic disease that is highly sensitive to chemotherapy [9,10]. Numerous studies have demonstrated that chemotherapy may prolong the disease-free survival of patients with localized rhabdomyosarcoma by approximately 70% [11]. At present, adjuvant chemotherapy has become a routine in the management of rhabdomyosarcoma. Two regimens have been recommended. While the VAC regimen is widely used worldwide, the IVA regimen is predominantly adopted in European countries.

Despite various treatment regimens, complete resection cannot be always attainable, particularly pelvic tumor cases. In patients with unresectable lesions, initial chemotherapy has a high response rate [12]. Nevertheless, surgery should be considered whenever possible after the initial chemotherapy since multiple studies have previously showed complete resection with negative margins after preoperative chemotherapy. Moreover, it has been documented that addition of neo-adjuvant chemotherapy may help preserve normal anatomy, improve bladder function, and increase the overall survival in genitourinary rhabdomyosarcoma patients [13,14]. In our study, almost all patients received adjuvant chemotherapy, a partial response (PR) was achieved in 5 patients who received neo-adjuvant chemotherapy, and the overall response rate was 100%. In all cases, the lesions became a surgically resectable disease, and complete excision was achieved. Nevertheless, the efficacy, toxicity and optimal dose, and time of neo-adjuvant chemotherapy warrant further more large-scale studies.

Clinical outcomes of the patients who were initially diagnosed with a metastasis disease were poor in this study. During the last 30 years, introduction of chemotherapy and radiation into the treatment of nonmetastatic rhabdomyosarcoma has improved the 3-year survival to 86% [15], which is in a sharp contrast to 30% for patients with a metastatic disease [16]. Treatment of metastatic rhabdomyosarcoma remains a significant challenge to oncologists. Although high-dose chemotherapy with stem cell rescue in a series of studies has achieved a high objective response rate, the ultimate outcome of patients with metastatic rhabdomyosarcoma remains unsatisfactory [17,18]. Therefore, considerable research and clinical efforts have been devoted to the development of new therapeutic strategies for metastatic rhabdomyosarcoma, and novel agents have showed potential anticancer activities in clinical trials. Irinotecan, a topoisomerase I inhibitor, has shown therapeutic potential for patients with rhabdomyosarcoma after failure with multidrug chemotherapy in preclinical studies and phase I trials [19,20]. In a further phase II trial, when irinotecan was used alone, it achieved a response rate of 11.4% in patients with recurrent or refractory rhabdomyosarcoma with a median time to progression of 1.4 years [21]. For newly diagnosed metastatic rhabdomyosarcoma, the response rate to irinotecan reached 42% to 45%. When vincristine and irinotecan were combined for relapsed rhabdomyosarcoma, the best overall objective response rate was 31.5%, and the median survival time was 1.4 years. When vincristine and irinotecan were used for newly diagnosed metastatic rhabdomyosarcoma, the response rate was 70%, and the survival rate could reach 71% [21]. Irinotecan is highly active against metastatic rhabdomyosarcoma and has attracted further investigations. Gee *et al.* have reported that vascular endothelial growth factor (VEGF) receptors exist in embryonal rhabdomyosarcoma where VEGF binding to VEGR receptors eventually promotes tumor cell proliferation [22]. Based on a previous retrospective study, rhabdomyosarcoma patients with high circulating levels of VEGF have poor survival [23]. In xenograft models of rhabdomyosarcoma, bevacizumab, an anti-VEGF mAb, has been demonstrated to be capable of inhibiting tumor growth and metastases and enhancing tumor sensitivity to radiation [24,25], thus possessing a therapeutic potential for refractory rhabdomyosarcoma. Ola Lindén reported an embryonal rhabdomyosarcoma case where the chemorefractory disease became resectable after radiotherapy in addition to treatment with bevacizumab and statins, and no progression occurred following postoperative adjuvant chemotherapy plus bevacizumab for 7 cycles [26]. In another study, compassionate use of bevacizumab in concomitance with topotecan on refractory or recurrent rhabdomyosarcoma resulted in a partial remission [27]. As demonstrated in a phase I study, bevacizumab is well-tolerated in children [28]. Currently, a phase II study is ongoing to evaluate the efficacy and safety of bevacizumab combined with chemotherapy for childhood and adolescent metastatic rhabdomyosarcoma.

A better understanding of molecular pathways involved in cancer development would identify potential therapeutic targets. Cumulating evidence has showed that insulin-like growth factor (IGF) signaling plays an important role in survival of patients with embryonal rhabdomyosarcoma [29,30], and thus, humanized monoclonal antibodies and small kinase inhibitors have been evaluated in many preclinical studies [31,32]. At present, a study sponsored by COG is underway to evaluate IMC-A12 and a monoclonal anti-IGF-IR antibody as a combination therapy for metastatic rhabdomyosarcoma. Moreover, it has been shown that epidermal growth factor receptor (EGFR) is highly expressed in embryonal rhabdomyosarcoma cells and thus may serve as a candidate therapeutic target [33]. Several studies, indeed, have shown that anti-EGFR-mediated neutralization of EGFR may result in effective growth inhibition of embryonal rhabdomyosarcoma cell lines *in vitro* [34,35]. It is highly anticipated that targeting molecular pathways will become a novel therapeutic for embryonal rhabdomyosarcoma.

In our study, the very 1 patient who presented with a progressive disease failed to respond to either a two-chemotherapy regimen or radiotherapy but achieved an exciting outcome after treatment with topotecan and bevacizumab in combination; the tumor size was reduced over 30%, and during the 11-month follow-up, no evident relapse was observed. It appears that bevacizumab is helpful in the management of relapsed chemorefractory rhabdomyosarcoma.

There are two staging systems for rhabdomyosarcoma. The Intergroup Rhabdomyosarcoma Study (IRS) has been using the clinical grouping (CG) system since 1972. This system stages rhabdomyosarcoma based on the extent of resection but does not include important prognostic factors, such as tumor size and lymph node involvement [36]. The other system is tumor-node-metastasis (TNM) staging system which classifies the cancer by its primary site, tumor size, regional node involvement, and the presence or absence of distant metastasis. Now, the TNM system is widely accepted worldwide [37]. In this system, tumor site is viewed as a prognostic factor. Unfavorable anatomic sites of the genitourinary system are the prostate and the bladder whereas favorable anatomic sites include the paratesticule and the vagina [38]. Results from the International Society of Pediatric Oncology (SIOP) and the IRS group showed that non-bladder/prostate genitourinary rhabdomyosarcoma has a better 5-year survival than bladder/prostate rhabdomyosarcoma [39]. In our study, the overall survival of bladder/prostate rhabdomyosarcoma was worse than that of non-bladder/prostate cases. However, the difference did not reach a statistically significant level, probably due to the limited number of patients.

Several previous studies have demonstrated that age is a prognostic factor for patients with rhabdomyosarcoma. A study analyzing IRSG data showed that children aged between 1 and 10 years had a better survival rate compared to those older than 10 years [40]. A retrospective analysis of 2,600 rhabdomyosarcoma cases showed that adults had a worse outcome than children, and the 5-year overall survival was 27% for adults but 61% for children [41]. Rhabdomyosarcoma appears to occur in unfavorable sites more frequently in adults. However, another retrospective study involving cases from a single institution indicated that the survival rate in adult rhabdomyosarcoma patients was not significantly lower than that in children with rhabdomyosarcoma if similar treatments were applied [42]. In addition, a report from the IRS-IV suggested that age did not affect survival in patients with a metastatic disease [16]. In our study, for the same pathological subtype, the survival rate was not significantly different between children aged under 10 years and those aged over 10 years. Moreover, the outcome in younger patients was worse than that in older patients. Thus, pathological types and therapeutic effects should be taken into consideration in further studies.

It has to be pointed out that patients with anemia had a shorter survival; the median survival was only 9 months and 1 year-survival rate was close to 0. Among the patients analyzed, 3 presented with chronic bleeding and 1 with bone marrow involvement. According to the current literature, bone marrow metastasis is not uncommon, and the incidence of bone marrow involvement at diagnosis varies from 29% to 50% [43]. Early diagnosis of bone marrow involvement is difficult because of the lack of specific clinical manifestations. Abnormal changes in complete blood count such as anemia, neutropenia or leukocytosis, and thrombocytopenia occur in some patients with bone marrow involvement [44]. According to a report from the IRS, although patients with bone marrow metastasis have a high response rate to the VAC regimen, the best survival time is seldom beyond 4 years [45]. In our study, 1 patient with advanced prostate embryonal rhabdomyosarcoma with hemoglobin (Hb) 65 g/L was diagnosed with bone marrow metastasis. Abdomen CT and MRI findings showed enlarged retroperitoneal lymph nodes and a primary mass of 8.5 cm in diameter protruding into the inferior part of the bladder. This patient underwent 8 cycles of chemotherapy with three different protocols but showed no response even to vincristine plus irinotecan. The median TTP was 3 months, and the overall survival time was 21 months. Patients with anemia at diagnosis were associated with large tumor mass, concomitant metastasis to lymph nodes and lungs and thus had poor prognosis.

The incidence of genitourinary embryonal rhabdomyosarcoma is quite low in China, the current study is a single-center retrospective study with maximum Chinese cases so far based on my knowledge, so I think this will bring a valuable knowledge to this field. Compared to the documents from other countries, Chinese patients in our institute received a similar treatment with the same guideline but have the lower survival rate compared to western countries. We preliminarily guess that is because of race difference but needs further detailed research.

This study has several limitations. First, this was a retrospective study with a small sample size. Second, the patients analyzed were treated by different surgeons. Without a standardized protocol, a surgeon’s experience might affect patient outcome. Third, in the analyzed cases, VAC was used as the neo-adjuvant chemotherapy regimen. However, there were no unified guidelines on neo-adjuvant treatment, and thus, the treatment dose and duration and the number of treatment cycles were not optimized. Fourth, the data on drug toxicities were incomplete. Despite these limitations, this study for the first time have characterized the clinicopathologic features and evaluated the treatment outcomes of genitourinary embryonal rhabdomyosarcoma in a sample of Chinese patients.

## Conclusions

Multimodality treatment including surgical operation, chemotherapy, and radiotherapy has gradually become the primary treatment for genitourinary embryonal rhabdomyosarcoma. For unresectable disease, neo-adjuvant chemotherapy plays an important role in maximizing the preservation of a functional bladder, increasing resection rates and improving the quality of life. However, metastatic disease with unfavorable prognosis has limited treatment choices and warrants development of novel therapeutic approaches. The findings in this study also support that the prognosis of genitourinary embryonal rhabdomyosarcoma is not necessarily poorer in older than in younger patients.

### Consent

Written informed consent was obtained from the patient for the publication of this report and any accompanying images.
